# Cancer mortality-to-incidence ratio as an indicator of cancer management outcomes in Organization for Economic Cooperation and Development countries

**DOI:** 10.4178/epih.e2017006

**Published:** 2017-02-05

**Authors:** Eunji Choi, Sangeun Lee, Bui Cam Nhung, Mina Suh, Boyoung Park, Jae Kwan Jun, Kui Son Choi

**Affiliations:** 1 Graduate School of Cancer Science and Policy, National Cancer Center, Goyang, Korea; 2 National Cancer Control Institute, National Cancer Center, Goyang, Korea

**Keywords:** Neoplasms, Early detection of cancer, Incidence, Mortality, Delivery of health care

## Abstract

**OBJECTIVES:**

Assessing long-term success and efficiency is an essential part of evaluating cancer control programs. The mortality-to-incidence ratio (MIR) can serve as an insightful indicator of cancer management outcomes for individual nations. By calculating MIRs for the top five cancers in Organization for Economic Cooperation and Development (OECD) countries, the current study attempted to characterize the outcomes of national cancer management policies according to the health system ranking of each country.

**METHODS:**

The MIRs for the five most burdensome cancers globally (lung, colorectal, prostate, stomach, and breast) were calculated for all 34 OECD countries using 2012 GLOBOCAN incidence and mortality statistics. Health system rankings reported by the World Health Organization in 2000 were updated with relevant information when possible. A linear regression model was created, using MIRs as the dependent variable and health system rankings as the independent variable.

**RESULTS:**

The linear relationships between MIRs and health system rankings for the five cancers were significant, with coefficients of determination ranging from 49 to 75% when outliers were excluded. A clear outlier, Korea reported lower-than-predicted MIRs for stomach and colorectal cancer, reflecting its strong national cancer control policies, especially cancer screening.

**CONCLUSIONS:**

The MIR was found to be a practical measure for evaluating the long-term success of cancer surveillance and the efficacy of cancer control programs, especially cancer screening. Extending the use of MIRs to evaluate other cancers may also prove useful.

## INTRODUCTION

Cancer is a leading cause of death in both more and less economically developed countries. In 2012, there were 14.1 million new cancer cases and 8.2 million cancer deaths worldwide; 57% (8 million) of new cancer cases and 65% (5.3 million) of cancer deaths occurred in less developed regions [1]. Due to population growth and aging, the global cancer burden is expected to grow. The five most common cancers (lung, breast, colorectal, prostate, and stomach) in both sexes account for nearly half of all cancer cases. Lung and breast cancer are the most frequently diagnosed cancers, and are the leading causes of cancer death in men and women, respectively, both overall and in less developed countries [2]. In general, cancer incidence rates are higher in more developed regions with longer life expectancies. In fact, the incidence rates for all cancers combined are twice as high for more developed countries than for less developed countries. However, mortality rates for all cancers are only 8 to 15% higher in more developed countries [2]. This disparity primarily reflects differences in the distribution of cancer cases, which is affected by risk factors, detection practices, and/or the availability of treatment.

A substantial portion of cancer cases and deaths could be prevented by broadly applying effective prevention measures, such as tobacco control, vaccination, and the use of early detection tests. Thus, the implementation of cancer control programs has been recommended as a means to effectively reduce cancer incidence and mortality, and national cancer control programs have been developed in several countries [3]. Nonetheless, assessing the long-term success and efficiency of these programs is essential. The mortality-to-incidence ratio (MIR) provides an alternative means to assess the burden of a disease by presenting mortality after accounting for incidence. In prior studies, the MIR was found to be a simple and insightful measure of the efficacy of cancer control programs [4,5]. The ratio identifies whether a country has a higher or lower mortality for a condition, normalized to its incidence. To determine the causes of differences in mortality and incidence, other information should be gathered. Previously, the MIR statistic has been used to demonstrate racial disparities in cancers [6], as well as to examine relationships between health care systems and cancer outcomes in the US [7] and worldwide [8]. Recently, Sunkara & Hébert [8] demonstrated a strong association between MIRs for colorectal cancer and the quality of health care systems. They suggested that the MIR could be useful as an indicator for identifying disparities in cancer screening and treatment internationally.

Therefore, in this study, we calculated MIRs for the five most common cancers in the 34 Organization for Economic Cooperation and Development (OECD) member countries in an attempt to evaluate the outcomes of national cancer management policies according to the performance of each country’s health system. Only OECD member countries were chosen because of their high-quality health care-related data. In particular, this study aimed to assess the outcomes of cancer control programs in Korea, as reflected by the MIR, in comparison to MIR values and health care system rankings across OECD countries. Additionally, we attempted to identify factors that could potentially explain outliers, in which MIRs were not well predicted by regression models.

## MATERIALS AND METHODS

### Mortality and incidence rate data

Mortality and incidence rate data were derived from the GLOBOCAN 2012 database for all 34 OECD countries [1]. The GLOBOCAN database provides contemporary estimates of the incidence, mortality, and prevalence of major types of cancer at the national level for 184 countries throughout the world. We collected the age-standardized rates per 100,000 population per year for lung, colorectal, prostate, stomach, and breast cancer, and calculated the MIRs by dividing the mortality rate by the incidence rate. When using the GLOBOCAN data, it is recommended to report the scope of the data sources and methods. In that database, the quality of the data on the incidence rate is graded from A (high quality) to G (no data), depending on the availability of incidence data. For grade G countries, GLOBOCAN contains estimated incidence rates using those of neighboring countries or registries in the same area [1]. Similarly, for mortality rates, data are scored from 1 (high quality, complete registration) to 6 (no data). In our data set of OECD countries, the mean grade of incidence rate data was grade B; only data for Greece and Hungary were given a grade of G. The mean score for the available mortality data was 1.79 (from 1 to 6), with Mexico reporting the highest score of five. Despite the poor quality of incidence or mortality data from Greece, Hungary, and Mexico, the methods used to estimate cancer incidence and mortality are well established and reported in the GLOBOCAN database. We therefore included all these countries in the analysis, in order to compare MIRs for the five most common cancers across the 34 OECD member countries. Moreover, the results were very similar, whether or not we included data from countries with poor-quality data (Mexico, Hungary, and Greece) in the analysis.

### Health system rankings

As an indicator of the quality of health systems, we adopted the health system rankings presented by the World Health Organization (WHO) in the year 2000 for 191 countries [9]. The health system ranking reflects five composite measures: overall health, health care financing, health inequality, health responsiveness, and distribution of health care services. Data for the five composite measures were derived from estimates for each country in 1997. Although the rankings have not been updated due to criticisms about their efficacy [10], we valued the methodological framework and the thoroughness of the data based on which the indicators were developed [11]. We decided to use the rankings after updating the composite measures when possible. Among the five composite measures, it was only possible to update overall health and health inequality. As overall health in the initial health ranking report was represented by health-adjusted life expectancy (HALE), we updated the measure with 2012 HALE data [12]. Health inequality was derived from calculations on child mortality [11]; therefore, we adopted 2011 OECD health data on child mortality. The other three measures (health care financing, health responsiveness, and distribution of health care services) were not able to be updated due to inconsistencies in the data available for all OECD countries. Our updated version of the health system rankings reflected rankings similar to those originally reported, except for Austria, Portugal, Greece, Switzerland, Canada, Australia, New Zealand, and Korea.

### Statistical analysis

A simple linear regression model was generated by taking the MIR as a dependent variable and the updated health system rankings as the independent variable. For the analysis, not the exact values of each health system ranking for countries, but the ranking number itself was used for the analysis, as previous studies have confirmed the presence of a linear association between the MIR and the health system ranking itself [8]. The formula for this analysis was as follows: predicted MIR= health system ranking× beta+alpha. Divergent points were then identified. Divergent points were defined as countries for which the residuals between their actual MIR and their predicted MIR determined by the regression model were greater or less than 0.07. After defining divergent points, we performed an additional simple linear regression analysis excluding divergent countries. Statistical analyses were performed using Stata version 13 (StataCorp., College Station, TX, USA), taking p-values < 0.05 to indicate statistical significance.

### Ethical issues

This study was exempted from the institutional ethics review board, because it was not human-subjects research and analyzed existing data.

## RESULTS

**Figure 1.**
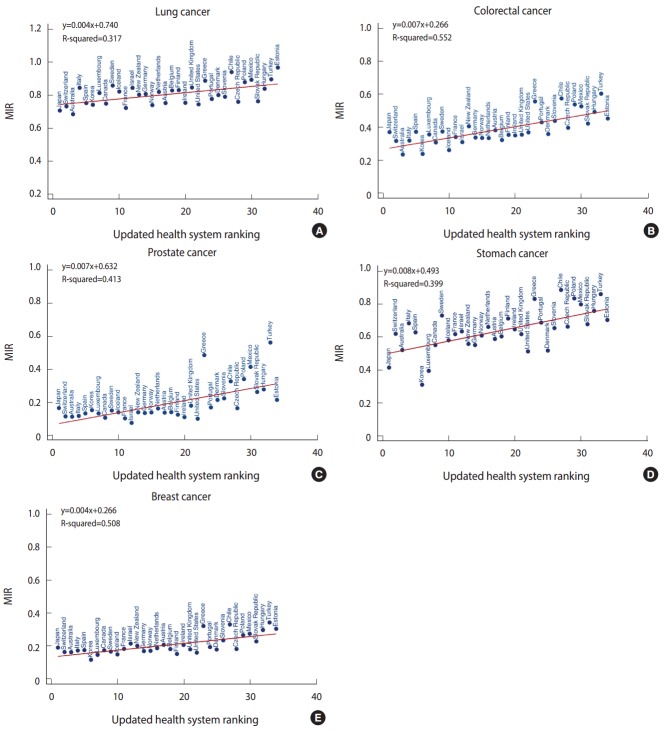
Mortality-to-incidence ratio (MIR) for the five most common cancers (A: lung, B: colorectal, C: prostate, D: stomach, and E: breast) vs. health system ranking for all 34 Organization for Economic Cooperation and Development countries.

Figure 1 (A-E) depicts scatter plots with predicted lines for lung, colorectal, prostate, stomach, and breast cancer. For all scatter plots, we detected significant linear relationships between the MIR and the health system rankings, with coefficients of determination ranging from 32 to 55%. These results demonstrated a positive association between lower health care system rankings (1-unit changes) and higher MIRs.

For lung cancer (Figure 1A), with every 1-unit change in health system ranking, there was a 0.004 increment rise in the MIR. Eight countries were identified as divergent points in the lung cancer model: the Slovak Republic, Czech Republic, US, and Australia demonstrated lower MIRs than predicted, whereas Sweden, Italy, Chile, and Estonia showed higher MIRs. Figure 1B presents a 0.007 incremental change in MIR for colorectal cancer with a 1-unit change in the health system ranking. Divergent points for this model included Denmark, Iceland, Korea, and Belgium, all of which had lower MIRs than predicted, and Spain, Poland, Japan, Turkey, Chile, and Greece, which had higher MIRs. In the prostate cancer model, a 1-unit change in health system ranking generated an increase in MIR of 0.007 units (Figure 1C). The US, Czech Republic, Ireland, Estonia, Finland, Israel, and Portugal had lower MIRs than predicted, while Chile, Japan, Mexico, Greece, and Turkey had higher MIRs than predicted in this model. For stomach cancer (Figure 1D), every 1-unit change in health system ranking led to an increase in MIR of 0.008 units. Its divergent points corresponding to a lower-than-predicted MIR were Korea, Denmark, the United States, Czech Republic, Luxembourg, Japan, Slovak Republic, and Estonia; higher-than-predicted MIRs were found for Spain, Turkey, Poland, Switzerland, Greece, Italy, Sweden, and Chile. Finally, for breast cancer (Figure 1E), a 1-unit change in health system ranking increased the MIR by 0.004 units. Among the divergent points for breast cancer, the Czech Republic exhibited a lower MIR, while Turkey, Chile, and Greece showed higher MIRs than predicted. Appendices 1-5 present the complete data on the updated health system rankings, mortality rates, incidence rates, actual MIRs, predicted MIRs, and residuals, alphabetically sorted by country name.

To eliminate the effect of divergent points, we excluded countries with residuals between their actual MIR and their predicted MIR that were greater or less than 0.07. Table 1 lists the coefficients of determination for the original model and the additional model devised after eliminating the divergent points. The R^2^ value for lung cancer in the original model was 0.32 (meaning that 32% of the total variability in MIR for lung cancer was explained by the model), and it increased to 0.49 after removing outliers. The R^2^ value for colorectal cancer increased from 0.55 to 0.68; the R^2^ value for prostate cancer increased from 0.41 to 0.75; the R^2^ value for stomach cancer increased from 0.40 to 0.73; and the R^2^ value for breast cancer increased from 0.51 to 0.55.

## DISCUSSION

In the present study, we demonstrated a significant positive linear relationship between the MIR and the updated health care system rankings. After removing divergent points, we detected substantial increases in the coefficients of determination for each cancer model, up to 0.75 for prostate cancer, meaning that 75% of the total variability in the MIR across countries was explained by the updated health care system rankings. In the lung cancer model, however, the coefficient of determination remained only at 0.49. Despite improvements in cancer treatment, the overall survival rate for lung cancer remains around 20% [13]. Additionally, although lung cancer screening with low-dose computed tomography is now recommended in several guidelines, researchers have yet to alleviate concerns about the sensitivity of the test [14]. Therefore, differences in the MIR for lung cancer among OECD countries might not be clearly explained by differences in health systems.

In the models for stomach and colorectal cancer, Korea was a clear divergent point, with MIRs that were much lower than predicted. While the average MIR among all OECD countries was 0.63 for stomach cancer, Korea reported an MIR of 0.31. In the colorectal cancer model, Korea’s MIR was 0.23, compared to the average MIR of 0.38. We suspect that the low MIRs for Korea reflect the nation’s strong national cancer control policies. In Korea, cancer is responsible for one in every four deaths [15]. In an effort to reduce the increasing cancer burden, the Korean government has supported cancer screening via the National Cancer Screening Program (NCSP) for the Korean population since 2002. Via the NCSP, medical aid enrollees and the lower 50% of income bracket among the National Health Insurance (NHI) beneficiaries are eligible for free-of-charge screening for stomach, breast, cervix, liver, and colorectal cancer. The more affluent 50% of NHI beneficiaries are eligible for screening with a co-payment of 10%. For detecting stomach cancer, eligible participants over the age of 40 years are invited biennially to undergo screening via upper endoscopy or upper gastrointestinal series. The total screening rate for stomach cancer was 73.6% in 2013 [16]. For colorectal cancer, individuals over 50 years of age are annually invited to undergo an initial mass screening with a fecal occult blood test, and a further examination with colonoscopy or double-contrast barium enema is provided for those with positive results. The screening rate for colorectal cancer was 55.6% in 2013 [16]. According to our results, we suggest that the nationwide cancer screening program in Korea appears to be associated with an MIR lower than that predicted by the regression model.

Similar implications are also applicable for other divergent points in the regression models. In Japan, stomach cancer is a serious burden, accounting for 14.2% of all cancer deaths [17]. To reduce this burden, Japan has also conducted stomach cancer screening with photofluorography as part of a national program. Under the national health policy for the prevention of chronic diseases, stomach cancer screening has been promoted by providing financial support for cancer screenings. In the present study, Japan showed a lower-than-predicted MIR for stomach cancer of 0.41. In contrast, the higher-than-predicted MIRs among divergent nations may stem from a lack of appropriate cancer control programs. For example, Chile, which also reports one of the highest incidence rates of stomach cancer, lacks screening guidelines for stomach cancer, though it has implemented a national integrated non-communicable disease policy and action plans [8]. Likewise, for colorectal cancer, Denmark, Iceland, and Belgium showed lower-than-predicted MIRs, and all have formal screening recommendations for colorectal cancer in place [18]. Meanwhile, countries with higher-than-predicted MIRs were less likely to have formal screening recommendations or tended to have lower screening rates for colorectal cancer [8].

Unexpectedly, Korea was not classified as a divergent nation in the breast cancer model, though it has provided biennial breast cancer screening with a mammography for all women over 40 years under the NCSP. For breast cancer, the majority of OECD countries conduct mammography screenings, with relatively high screening rates. In addition, the treatment of breast cancer has improved greatly with the introduction of multidisciplinary breast cancer care units, reducing the benefits from mammography screening. Still, Korea reported a lower actual MIR of 0.11 for breast cancer than its predicted MIR of 0.15, which is also lower than the average MIR across OECD countries of 0.20.

The NCSP in Korea does not provide nationwide screening for lung and prostate cancer. Nevertheless, the nation still recorded an actual MIR for lung cancer of 0.74, lower than its predicted value of 0.76 and lower than the average value for all OECD countries of 0.80. This might be explained by Korea’s comparatively high 5-year survival rates for lung cancer. Korea had a 5-year survival rate for lung cancer of 20.7%, while the 5-year survival rates were 16.6% in the US, 17% in Canada, and 29.7% in Japan [15,19,20]. For prostate cancer, Korea reported a higher actual MIR of 0.15 than the predicted value of 0.11. In comparison, the actual MIR for prostate cancer in the US was 0.10, the lowest among all OECD countries. In the US, prostate cancer is the most common cancer and the second leading cause of death among men, according to the National Cancer Institute statistics [21]. To the reduce cancer burden, prostate cancer screening is recommended by the American Cancer Society with informed consent, although the US Preventive Services Task Force has warned against prostate cancer screening because its harms may outweigh its benefits. Nevertheless, the guidelines and screening programs for prostate cancer proposed by the American Cancer Society seem to have helped effectively control prostate cancer, as reflected by its low MIR [22].

Our study has several limitations that warrant consideration. First, our data focused wholly on OECD countries, which generally have more sound health infrastructure. This limits the generalizability of our results to low-income and middle-income countries lacking the needed infrastructure. Second, there were inconsistencies in the data sources and methods for determining cancer mortality and incidence rates from GLOBOCAN, as described in the Methods section. Nonetheless, our findings were consistent regardless of whether we included data from countries with poor-quality data. Furthermore, updating the data for the WHO 2000 health system rankings was not fully achieved due to a lack of available data. Thus, our rankings may not exactly reflect the most recent performance of each nation’s health care system.

In this study, we found that lower MIRs reflected the implementation of effective cancer control programs, including cancer screening. In contrast, countries with higher-than-predicted MIRs often lacked proper health policies or recommendations for cancer control. For Korea, among the five cancers analyzed in this study, stomach and colorectal cancer had markedly low MIRs, indicating effective cancer control, mainly as a result of screening programs offered via the NCSP. Despite finding the MIR to be an efficient and useful indicator of cancer control outcomes, studies on mortality rate reductions are required to confirm the effectiveness of cancer control. Notwithstanding, we favor extending the use of the MIR for other cancers to assess the long-term success of cancer screening programs.

## Figures and Tables

**Table 1.** Coefficients of determination before and after removing outliers

Cancer	R^2^ in original models	R^2^ without outliers
Lung	0.32	0.49
Colorectal	0.55	0.68
Prostate	0.41	0.75
Stomach	0.40	0.73
Breast	0.51	0.55

## Appendix 1.

Raw data and regression results for lung cancer based on MIRs for all OECD countries

**Table:**

Country	Updated HSR	Mortality rate	Incidence rate	Actual MIR	Regression-predicted MIR	Residual
Australia	3	18.5	27.0	0.68	0.75	-0.07
Austria	17	20.7	27.5	0.75	0.80	-0.05
Belgium	18	30.5	36.8	0.83	0.81	0.02
Canada	8	28.4	37.9	0.75	0.77	-0.02
Chile	27	12.5	13.3	0.94	0.84	0.10
Czech Republic	28	24.7	32.5	0.76	0.84	-0.09
Denmark	25	31.4	39.2	0.80	0.83	-0.03
Estonia	34	23.6	24.4	0.97	0.87	0.10
Finland	19	16.7	20.1	0.83	0.81	0.02
France	11	25.3	35.0	0.72	0.78	-0.06
Germany	14	22.2	27.5	0.81	0.79	0.01
Greece	23	25.3	28.5	0.89	0.83	0.06
Hungary	32	43.3	51.6	0.84	0.86	-0.02
Iceland	10	24.5	29.8	0.82	0.78	0.04
Ireland	20	23.6	31.3	0.75	0.81	-0.06
Israel	12	17.9	21.2	0.84	0.78	0.06
Italy	4	20.7	24.5	0.84	0.75	0.09
Japan	1	17.4	24.6	0.71	0.74	-0.04
Korea	6	21.3	28.7	0.74	0.76	-0.02
Luxembourg	7	23.1	28.4	0.81	0.77	0.05
Mexico	30	6.7	7.5	0.89	0.85	0.04
Netherlands	16	30.5	37.2	0.82	0.80	0.02
New Zealand	13	20.8	25.9	0.80	0.79	0.01
Norway	15	22.2	30.0	0.74	0.80	-0.06
Poland	29	33.4	38.0	0.88	0.85	0.03
Portugal	24	15.7	20.2	0.78	0.83	-0.05
Slovak Republic	31	21.6	28.3	0.76	0.86	-0.09
Slovenia	26	26.8	33.9	0.79	0.84	-0.05
Spain	5	22.8	30.3	0.75	0.76	-0.01
Sweden	9	16.4	19.1	0.86	0.77	0.08
Switzerland	2	20.0	27.3	0.73	0.75	-0.01
Turkey	33	31.1	34.7	0.90	0.86	0.03
United Kingdom	21	25.4	30.0	0.85	0.82	0.03
United States	22	28.6	38.4	0.74	0.82	-0.08

MIR, mortality-to-incidence ratio; OECD, Organization for Economic Cooperation and Development; HSR, health system ranking.

## Appendix 2.

Raw data and regression results for colorectal cancer based on MIRs for all OECD countries

**Table:**

Country	Updated HSR	Mortality rate	Incidence rate	Actual MIR	Regression-predicted MIR	Residual
Australia	3	9.0	38.4	0.23	0.29	-0.05
Austria	17	9.9	26.0	0.38	0.38	0.00
Belgium	18	11.8	36.7	0.32	0.39	-0.07
Canada	8	10.8	35.2	0.31	0.32	-0.01
Chile	27	8.6	15.0	0.57	0.45	0.12
Czech Republic	28	15.4	38.9	0.40	0.46	-0.06
Denmark	25	14.5	40.5	0.36	0.44	-0.08
Estonia	34	12.3	27.2	0.45	0.50	-0.05
Finland	19	8.3	23.5	0.35	0.40	-0.04
France	11	10.2	30.0	0.34	0.34	0.00
Germany	14	10.4	30.9	0.34	0.36	-0.03
Greece	23	7.5	13.5	0.56	0.42	0.13
Hungary	32	20.8	42.3	0.49	0.48	0.01
Iceland	10	7.4	28.4	0.26	0.33	-0.07
Ireland	20	12.2	34.9	0.35	0.40	-0.05
Israel	12	11.1	35.9	0.31	0.35	-0.04
Italy	4	10.8	33.9	0.32	0.29	0.03
Japan	1	11.9	32.2	0.37	0.27	0.10
Korea	6	10.7	45.0	0.24	0.31	-0.07
Luxembourg	7	11.2	31.5	0.36	0.31	0.04
Mexico	30	4.1	7.8	0.53	0.47	0.05
Netherlands	16	13.4	40.2	0.33	0.37	-0.04
New Zealand	13	15.1	37.3	0.40	0.35	0.05
Norway	15	13.0	38.9	0.33	0.37	-0.03
Poland	29	14.5	27.0	0.54	0.46	0.07
Portugal	24	13.6	31.7	0.43	0.43	0.00
Slovak Republic	31	18.0	42.7	0.42	0.48	-0.06
Slovenia	26	16.2	37.0	0.44	0.44	-0.01
Spain	5	12.3	33.1	0.37	0.30	0.07
Sweden	9	10.9	29.2	0.37	0.33	0.05
Switzerland	2	9.3	29.4	0.32	0.28	0.04
Turkey	33	10.0	16.6	0.60	0.49	0.11
United Kingdom	21	10.7	30.2	0.35	0.41	-0.06
United States	22	9.2	25.0	0.34	0.42	-0.05

MIR, mortality-to-incidence ratio; OECD, Organization for Economic Cooperation and Development; HSR, health system ranking.

## Appendix 3.

Raw data and regression results for prostate cancer based on MIRs for all OECD countries

**Table:**

Country	Updated HSR	Mortality rate	Incidence rate	Actual MIR	Regression-predicted MIR	Residual
Australia	3	12.9	115.2	0.11	0.08	0.03
Austria	17	10.2	74.7	0.14	0.19	-0.05
Belgium	18	12.7	90.9	0.14	0.19	-0.06
Canada	8	9.4	88.9	0.11	0.12	-0.02
Chile	27	17.1	52.4	0.33	0.26	0.07
Czech Republic	28	11.8	72.2	0.16	0.27	-0.10
Denmark	25	19.5	91.3	0.21	0.25	-0.03
Estonia	34	20.2	94.4	0.21	0.31	-0.10
Finland	19	12.0	96.6	0.12	0.20	-0.08
France	11	10.0	98.0	0.10	0.15	-0.04
Germany	14	10.4	77.3	0.13	0.17	-0.03
Greece	23	9.8	20.2	0.48	0.23	0.25
Hungary	32	10.4	37.5	0.28	0.30	-0.02
Iceland	10	14.8	106.6	0.14	0.14	0.00
Ireland	20	12.5	114.2	0.11	0.21	-0.10
Israel	12	6.3	84.3	0.07	0.15	-0.08
Italy	4	7.9	67.6	0.12	0.09	0.02
Japan	1	5.0	30.4	0.16	0.07	0.09
Korea	6	4.6	30.3	0.15	0.11	0.04
Luxembourg	7	10.4	78.8	0.13	0.11	0.02
Mexico	30	11.3	27.3	0.41	0.28	0.13
Netherlands	16	13.5	83.4	0.16	0.18	-0.02
New Zealand	13	12.8	92.2	0.14	0.16	-0.02
Norway	15	17.9	129.7	0.14	0.17	-0.03
Poland	29	12.2	35.9	0.34	0.27	0.06
Portugal	24	10.7	63.6	0.17	0.24	-0.07
Slovak Republic	31	13.1	50.0	0.26	0.29	-0.03
Slovenia	26	18.5	82.9	0.22	0.25	-0.03
Spain	5	8.6	65.2	0.13	0.10	0.03
Sweden	9	17.8	119.0	0.15	0.13	0.02
Switzerland	2	12.2	107.2	0.11	0.08	0.04
Turkey	33	22.8	40.6	0.56	0.30	0.26
United Kingdom	21	13.1	73.2	0.18	0.22	-0.04
United States	22	9.8	98.2	0.10	0.22	-0.12

MIR, mortality-to-incidence ratio; OECD, Organization for Economic Cooperation and Development; HSR, health system ranking.

## Appendix 4.

Raw data and regression results for stomach cancer based on MIRs for all OECD countries

**Table:**

Country	Updated HSR	Mortality rate	Incidence rate	Actual MIR	Regression-predicted MIR	Residual
Australia	3	2.5	4.8	0.52	0.52	0.00
Austria	17	4.0	6.8	0.59	0.63	-0.04
Belgium	18	3.5	5.8	0.60	0.64	-0.04
Canada	8	2.7	4.9	0.55	0.56	-0.01
Chile	27	13.8	15.6	0.88	0.71	0.17
Czech Republic	28	4.9	7.4	0.66	0.82	-0.16
Denmark	25	2.9	5.6	0.52	0.70	-0.18
Estonia	34	9.7	13.8	0.70	0.77	-0.07
Finland	19	3.7	5.2	0.71	0.65	0.06
France	11	2.9	4.7	0.62	0.58	0.03
Germany	14	4.3	7.8	0.55	0.61	-0.06
Greece	23	4.4	5.3	0.83	0.68	0.15
Hungary	32	7.2	9.5	0.76	0.75	0.00
Iceland	10	2.9	5.0	0.58	0.57	0.01
Ireland	20	4.2	6.5	0.65	0.66	-0.01
Israel	12	4.5	7.1	0.63	0.59	0.04
Italy	4	5.6	8.2	0.68	0.53	0.16
Japan	1	12.4	29.9	0.41	0.50	-0.09
Korea	6	13.0	41.8	0.31	0.54	-0.23
Luxembourg	7	3.0	7.6	0.39	0.55	-0.16
Mexico	30	5.5	6.9	0.80	0.74	0.06
Netherlands	16	3.7	5.6	0.66	0.62	0.04
New Zealand	13	2.9	5.2	0.56	0.60	-0.04
Norway	15	2.8	4.6	0.61	0.61	-0.01
Poland	29	7.0	8.4	0.83	0.73	0.10
Portugal	24	9.0	13.1	0.69	0.69	0.00
Slovak Republic	31	6.5	9.6	0.68	0.74	-0.07
Slovenia	26	6.8	10.4	0.65	0.71	-0.05
Spain	5	4.9	7.8	0.63	0.53	0.09
Sweden	9	2.7	3.7	0.73	0.57	0.16
Switzerland	2	2.6	4.2	0.62	0.51	0.11
Turkey	33	12.2	14.2	0.86	0.76	0.10
United Kingdom	21	2.9	4.7	0.62	0.66	-0.05
United States	22	2.0	3.9	0.51	0.67	-0.16

MIR, mortality-to-incidence ratio; OECD, Organization for Economic Cooperation and Development; HSR, health system ranking.

## Appendix 5.

Raw data and regression results for breast cancer based on MIRs for all OECD countries

**Table:**

Country	Updated HSR	Mortality rate	Incidence rate	Actual MIR	Regression-predicted MIR	Residual
Australia	3	14.0	86.0	0.16	0.15	0.02
Austria	17	14.1	68.0	0.21	0.20	0.00
Belgium	18	20.3	111.9	0.18	0.21	-0.03
Canada	8	13.9	79.8	0.17	0.17	0.01
Chile	27	11.5	34.8	0.33	0.24	0.09
Czech Republic	28	12.8	70.3	0.18	0.25	-0.07
Denmark	25	18.8	105.0	0.18	0.24	-0.06
Estonia	34	15.7	51.6	0.30	0.27	0.03
Finland	19	13.6	89.4	0.15	0.21	-0.06
France	11	16.4	89.7	0.18	0.18	0.00
Germany	14	15.5	91.6	0.17	0.19	-0.02
Greece	23	14.1	43.9	0.32	0.23	0.09
Hungary	32	16.2	54.5	0.30	0.26	0.03
Iceland	10	14.4	96.3	0.15	0.17	-0.03
Ireland	20	19.1	92.3	0.21	0.22	-0.01
Israel	12	17.3	80.5	0.21	0.18	0.03
Italy	4	15.8	91.3	0.17	0.15	0.02
Japan	1	9.8	51.5	0.19	0.14	0.05
Korea	6	6.1	52.1	0.12	0.16	-0.04
Luxembourg	7	13.1	89.1	0.15	0.16	-0.02
Mexico	30	9.7	35.4	0.27	0.26	0.02
Netherlands	16	18.5	99.0	0.19	0.20	-0.01
New Zealand	13	17.1	85.0	0.20	0.19	0.01
Norway	15	12.5	73.1	0.17	0.19	-0.02
Poland	29	13.8	51.9	0.27	0.25	0.01
Portugal	24	13.1	67.6	0.19	0.23	-0.04
Slovak Republic	31	13.1	57.5	0.23	0.26	-0.03
Slovenia	26	15.6	66.5	0.23	0.24	-0.01
Spain	5	11.8	67.3	0.17	0.15	0.02
Sweden	9	13.4	80.4	0.17	0.17	0.00
Switzerland	2	13.6	83.1	0.16	0.14	0.02
Turkey	33	13.4	39.1	0.34	0.27	0.07
United Kingdom	21	17.1	95.0	0.18	0.22	-0.04
United States	22	14.9	92.9	0.16	0.22	-0.06

MIR, mortality-to-incidence ratio; OECD, Organization for Economic Cooperation and Development; HSR, health system ranking.
